# Meta-Analysis of the Association between COX-2 Polymorphisms and Risk of Colorectal Cancer Based on Case–Control Studies

**DOI:** 10.1371/journal.pone.0094790

**Published:** 2014-04-14

**Authors:** Qiliu Peng, Shi Yang, Xianjun Lao, Weizhong Tang, Zhiping Chen, Hao Lai, Jian Wang, Jingzhe Sui, Xue Qin, Shan Li

**Affiliations:** 1 Department of Clinical Laboratory, First Affiliated Hospital of Guangxi Medical University, Nanning, Guangxi, China; 2 Department of Anal and Colorectal Surgery, First Affiliated Hospital of Guangxi Medical University, Nanning, Guangxi, China; 3 Department of Occupational Health and Environmental Health, School of Public Health at Guangxi Medical University, Nanning, Guangxi, China; 4 Department of Gastrointestinal Surgery, Tumor Hospital of Guangxi Medical University, Nanning, Guangxi, China; National University of Ireland Galway, Ireland

## Abstract

**Objective:**

Cyclooxygenase-2 (COX-2) is an inducible enzyme converting arachidonic acid to prostaglandins and playing important roles in inflammatory diseases as well as tumor development. Previous studies investigating the association between COX-2 polymorphisms and colorectal cancer (CRC) risk reported conflicting results. We performed a meta-analysis of all available studies to explore this association.

**Methods:**

All studies published up to October 2013 on the association between COX-2 polymorphisms and CRC risk were identified by searching electronic databases PubMed, EMBASE, and Cochrane library. The association between COX-2 polymorphisms and CRC risk was assessed by odds ratios (ORs) together with their 95% confidence intervals (CIs).

**Results:**

Ten studies with 6,774 cases and 9,772 controls were included for −1195A>G polymorphism, 13 studies including 6,807 cases and 10,052 controls were available for −765G>C polymorphism, and 8 studies containing 5,121 cases and 7,487 controls were included for 8473T>C polymorphism. With respect to −765G>C polymorphism, we did not find a significant association with CRC risk when all eligible studies were pooled into the meta-analysis. However, in subgroup analyses by ethnicity and cancer location, with a Bonferroni corrected alpha of 0.05/2, statistical significant increased CRC risk was found in the Asian populations (dominant model CC+CG vs. GG: OR = 1.399, 95%CI: 1.113–1.760, P = 0.004) and rectum cancer patients (CC vs. GG: OR = 2.270, 95%CI: 1.295–3.980, P = 0.004; Recessive model CC vs. CG+GG: OR = 2.269, 95%CI: 1.297–3.970, P = 0.004). In subgroup analysis according to source of control, no significant association was detected. With respect to −1195A>G and 8473T>C polymorphisms, no significant association with CRC risk was demonstrated in the overall and subgroup analyses.

**Conclusions:**

The present meta-analysis suggests that the COX-2 −765G>C polymorphism may be a risk factor for CRC in Asians and rectum cancer patients. Further large and well-designed studies are needed to confirm this association.

## Introduction

Colorectal cancer (CRC) is the second most commonly diagnosed cancer with over 1.2 million new cases and 608,700 deaths in 2008 [1], [2]. The highest incidence rate of CRC is found in Australia, Europe, and North America [2]. In addition, the incidence rate of CRC is rapidly increasing in a number of countries within Eastern Asia, such as China [2]. Though the exact mechanism of CRC is still unknown, it has been well accepted that smoking, obesity, red meat consumption, and excessive alcohol consumption are risk factors for CRC [3], [4]. However, most individuals exposing to these known risk factors never develop CRC while many CRC cases develop among individuals without those known risk factors, suggesting that other factors such as genetic factors also play an important role in the development of CRC.

Cyclooxygenase-2 (COX-2) is an inducible enzyme that converts arachidonic acid to prostaglandins, which are potent mediators of inflammation. Through the production of prostaglandins, COX-2 is widely regarded as pro-inflammatory factor which can be activated by cytokines, mitogens, and growth factors at both the transcriptional and post-transcriptional levels [5]. Besides, accumulating evidence shows that COX-2 may play a key role in tumorigenesis of a variety of human malignancies by stimulating cell proliferation, inhibiting apoptosis, stimulating angiogenesis, and mediating immune suppression [6], [7], [8], [9]. The human COX-2 gene, mapped to chromosome 1q25.2–q25.3, is 7.5 kb in length and contains 10 exons [10]. Several potentially functional single-nucleotide polymorphisms (SNP), −765G>C (reference SNP ID, rs20417), −1195G>A (rs689466), and 8473T>C (rs5275) in the COX-2 gene have been identified. It was reported that these three SNPs modulated the inflammatory response through affecting gene transcription and/or mRNA stability, and consequently contributed to individual variation in susceptibility to cancers [11]. CRC is a typical inflammation-related malignancy, the pathological progress of CRC is a chronic inflammatory process [12]. Hence, it is biologically reasonable to hypothesize a potential relationship between the COX-2 gene polymorphisms and CRC risk.

Over the last two decades, a number of molecular epidemiological studies have been conducted to investigate the association between COX-2 −765G>C, −1195G>A, and 8473T>C polymorphisms and CRC risk, but the results remain controversial and inconclusive. With respect to −765G>C polymorphism, a meta-analysis by Cao et al [13]. found that individuals carrying the GC+CC genotypes were associated with increased risk of CRC among Asians (OR = 1.40, 95%CI: 1.11–1.76), however, they failed to include the largest sample study by Markar et al. [14] and other eligible studies [15], [16], which might make their conclusions questionable. With respect to −1195G>A, and 8473T>C polymorphisms, to the best of our knowledge, no meta-analyses on this issue have ever appeared. To derive a more precise estimation of the relationship between COX-2 polymorphisms and CRC risk, we conducted a meta-analysis of all available case–control studies relating the −765G>C, −1195G>A, and 8473T>C polymorphisms of the COX-2 gene to the risk of developing CRC.

## Materials and Methods

### Search strategy

We conducted a comprehensive literature search in PubMed, Embase, and Cochrane library databases up to October 01, 2013 using the following search strategy: (“colorectal cancer”, “CRC”, “colon cancer” or “rectum cancer”) and (“cyclooxygenase-2”, “COX-2”, or PTGS2) and (“polymorphism”, “variation”, “mutation”, “genotype”, or “genetic polymorphism”). There was no restriction on time period, sample size, population, language, or type of report. All eligible studies were retrieved and their references were checked for other relevant studies. The literature retrieval was performed in duplication by two independent reviewers (Qiliu Peng and Xue Qin). When multiple publications reported on the same or overlapping data, we chose the most recent or largest population. When a study reported the results on different subpopulations, we treated it as separate studies in the meta-analysis. The study was performed according to the proposal of Meta-analysis of Observational Studies in Epidemiology group (MOOSE) [17].

### Selection criteria

The following criteria were used to include published studies: (1) Case–control studies which evaluated the association between COX-2 polymorphisms and CRC risk; (2) had an odds ratio (OR) with 95% confidence interval (CI) or other available data for estimating OR (95% CI); and (3) control population did not contain malignant tumor patients. Studies were excluded if one of the following existed: (1) the design was based on family or sibling pairs; (2) the genotype frequency was not reported; (3) there was insufficient information for data extraction; and (4) conference abstracts, case reports, editorials, review articles, and letters.

### Data extraction

Two reviewers (Qiliu Peng and Xianjun Lao) independently reviewed and extracted data from all eligible studies. To ensure the accuracy of the extracted information, the two investigators checked the data extraction results and reached consensus on all of the data extracted. If different results were generated, they would check the data again and have a discussion to come to an agreement. If these two authors could not reach a consensus, another author (Xue Qin) was consulted to resolve the dispute and a final decision was made by the majority of the votes. Data extracted from eligible studies included the first author, year of publication, country of origin, ethnicity, genotyping method, matching criteria, source of control, CRC diagnosis criteria, total numbers of cases and controls and genotype frequencies of cases and controls. Ethnic backgrounds were categorized as Caucasian, and Asian. When a study did not state the ethnic descendent or if it was not possible to separate participants according to such phenotype, the group reported was termed as “mixed ethnicity”.

### Statistical analysis

The strength of the association between COX-2 polymorphisms and CRC risk was measured by odds ratios (ORs) with 95% confidence intervals (CIs). The significance of the pooled OR was determined by Z test and a *p* value less than 0.05 was considered significant. The association of COX-2 polymorphisms with CRC risk was assessed using additive models, recessive model, and dominant model.

Heterogeneity in meta-analysis refers to the variation in study outcomes between different studies. We used the *Q* test and *I^2^* statistics to assess the statistical heterogeneity among studies [18], [19]. If the result of the *Q* test was *P_h_*>0.1 and *I^2^*<50%, indicating the absence of heterogeneity, then a fixed-effects model (the Mantel–Haenszel method) was used to estimate the summary ORs [20]; otherwise, the random-effects model (the DerSimonian and Laird method) was used [21]. To explore the sources of heterogeneity among studies, we performed logistic metaregression and subgroup analyses. The following study characteristics were included as covariates in the metaregression analysis: ethnicity (Caucasians versus Asians), source of controls (Hospital-based versus Population-based), genotyping methods (PCR-RFLP versus not PCR-RFLP), and CRC confirmation (pathologically or histologically confirmed versus other diagnosis criteria). Subgroup analyses were conducted by ethnicity, cancer location, source of control, and HWE in controls.

Sensitivity analysis was performed by sequential omission of individual studies. For each polymorphism, publication bias was evaluated using a funnel plot and Egger's regression asymmetry test [22]. If publication bias existed, the Duval and Tweedie non-parametric “trim and fill” method was used to adjust for it [23]. The Bonferroni correction method was used to adjust for multiple comparisons. The distribution of the genotypes in the control population was tested for HWE using a goodness-of-fit Chi-square test. All analyses were performed using Stata software, version 12.0 (Stata Corp., College Station, TX). All *p* values were two-sided. To ensure the reliability and the accuracy of the results, two authors entered the data into the statistical software programs independently with the same results.

## Results

### Study characteristics

Based on the search criteria, 19 studies relevant to the role of COX-2 polymorphisms on CRC susceptibility were identified. Five of these articles were excluded: two were based on family or sibling pairs [24], [25], two did not provide allele or genotyping data [26], [27], and one was a meta-analysis [13]. Manual search of references cited in the published studies did not reveal any additional articles. As a result, a total of 14 relevant studies met the inclusion criteria for the meta-analysis [14], [15], [16], [28], [29], [30], [31], [32], [33], [34], [35], [36], [37], [38] (Figure S1). Among them, two of the eligible studies contained data on two different ethnic groups, and we treated them independently [14], [31]. Therefore, a total of 16 separate comparisons were finally included in the meta-analysis. The main characteristics of the 16 case–control comparisons are summarized in Table 1. Among them, 13 studies including 6,807 cases and 10,052 controls were available for −765G>C polymorphism, 10 studies with 6,774 cases and 9,772 controls for −1195A>G polymorphism, and 8 studies containing 5,121 cases and 7,487 controls for 8473T>C polymorphism. The sample size of these studies varied considerably, ranging from 230 to 4,552 individuals. Of all the eligible studies, 9 were conducted in Caucasians and 4 were in Asians for −765G>C polymorphism; 8 were conducted in Caucasians and 2 were in Asians for −1195A>G polymorphism; all the 8 studies were conducted in Caucasians for 8473T>C polymorphism. Seven studies were population–based and 9 were hospital–based studies. Nine studies in the present meta-analysis did not provide definite criteria for the CRC confirmation. Several genotyping methods were used, including PCR-RFLP, TaqMan assay, PCR-CTTP, and Pyrosequencing™. The genotype distributions among the controls in two studies were not consistent with HWE for -1195A>G [16], [32].

**Table 1:** Characteristics of eligible studies.

First author (Year)	Ethnicity (Country)	Sample size (case/control)	Genotypingmethods	Matching criteria	Source of control	CRC confirmation	SNPs studied	HWE(*P* value)		
								−1195A>G	−765G>C	8473T>C
Hamajima2001	Asian (Japan)	148/241	PCR-CTTP	NA	HB	NA	−765G>C	—	0.717	—
Cox 2004	Caucasion (Spain)	290/271	TaqMan assay	Age and gender	HB	HC	−765G>C, 8473T>C	—	0.730	0.639
Koh 2004	Asian (Singapore)	310/1177	TaqMan assay	Drinking	PB	HC	−765G>C	—	0.430	—
Siezen 1 2006	Caucasion (Netherlands)	200/388	Pyrosequencing™	Age and gender	PB	NA	−1195A>G, 8473T>C	0.343	—	0.996
Siezen 2 2006	Caucasion (Netherlands)	442/693	Pyrosequencing™	Age and gender	PB	NA	−1195A>G, 8473T>C	0.149	—	0.198
Tan 2007	Asian (China)	1000/1300	PCR-RFLP	Age and gender	HB	HC	−1195A>G, −765G>C	**0.020**	0.371	—
Xing 2008	Asian (China)	137/199	PCR-RFLP	Age and gender	HB	HC	−765G>C	—	0.838	—
Hof 2009	Caucasion (Netherlands)	326/369	PCR-RFLP	Age and gender	HB	NA	−1195A>G, −765G>C	0.471	0.260	—
Thompson 2009	Caucasion (America)	422/480	TaqMan assay	Ethnicity	PB	NA	−1195A>G, −765G>C, 8473T>C	0.131	0.286	0.081
Andersen 2009	Caucasion (Denmark)	359/765	TaqMan assay	Gender	PB	NA	−1195A>G, −765G>C, 8473T>C	0.177	0.609	0.746
Iglesias 2009	Caucasion (Spain)	284/123	PCR-RFLP	Age and gender	HB	PC	−765G>C	—	0.480	—
Pereira 2010	Caucasion (Portugal)	117/256	PCR-RFLP	NA	HB	HC	−1195A>G, −765G>C, 8473T>C	0.634	0.373	0.638
Daraei 2012	Caucasion (Iran)	110/120	PCR-RFLP	Age,gender, BMI and smoking	HB	NA	−765G>C	—	0.201	—
Makar 1 2013	Caucasion (America)	2003/2549	TaqMan assay	Age and gender	PB	NA	−1195A>G, −765G>C, 8473T>C	0.200	0.126	0.392
Makar 2 2013	Caucasion (America)	1436/2344	TaqMan assay	Age and gender	PB	NA	−1195A>G, −765G>C, 8473T>C	0.467	0.313	0.713
Li 2013	Asian (China)	451/629	PCR-RFLP	Gender	HB	PC	−1195A>G	**0.045**	—	—

SNP, Single nucleotide polymorphism; HC, Histologically confirmed; PC, Pathologically confirmed; NA, Not available; PB, Population–based; HB, Hospital–based; HWE, Hardy–Weinberg equilibrium in control population; PCR–RFLP, Polymerase chain reaction-restriction fragment length polymorphism; PCR-CTTP, Polymerase chain reaction with confronting two-pair primers.

### Meta-analysis results

**Figure 1:**
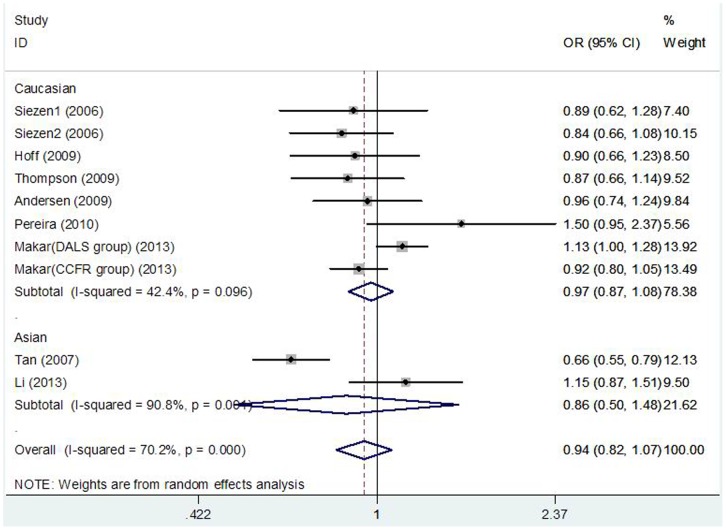
Forest plot of the COX-2 −1195G>A polymorphism and CRC risk using a random-effect model (dominant model GG+AG vs. AA).

Table 2 lists the main results of the meta-analysis of COX-2 -1195A>G polymorphism and CRC risk. There was no evidence of significant association between COX-2 -1195A>G polymorphism and CRC risk when all the eligible studies were pooled into the meta-analysis (GG vs. AA: OR = 0.902, 95% CI = 0.717–1.136, P = 0.380; AG vs. AA: OR = 0.945, 95% CI = 0.835–1.069, P = 0.369; GG+AG vs. AA: OR = 0.940, 95%CI = 0.822–1.074, P = 0.361, Figure 1; GG vs. AG+AA: OR = 0.891, 95%CI = 0.786–1.010, P = 0.072). In subgroup analyses by ethnicity, cancer location, source of control, and HWE in controls, statistical significant association was also not observed in all subgroups.

**Table 2:** Meta-analysis of COX-2 −1195A>G polymorphism and CRC risk.

Analysis	No. of studies	Homozygote (GG vs. AA)		Heterozygote (AG vs. AA)		Dominant model (GG+AG vs. AA)		Recessive model (GG vs. AG+AA)	
		OR (95% CI)	*P/P*_h_	OR (95% CI)	*P/P*_h_	OR (95% CI)	*P/P*_h_	OR (95% CI)	*P/P*_h_
Overall	10	0.902(0.717–1.136)	0.380/0.022	0.945(0.835–1.069)	0.369/0.004	0.940(0.822–1.074)	0.361/<0.001	0.891(0.786–1.010)	0.072/0.401
Ethnicity									
Caucasian	8	0.981(0.815–1.181)	0.843/0.833	0.991(0.918–1.069)	0.806/0.118	0.971(0.871–1.083)	0.598/0.096	0.986(0.820–1.185)	0.881/0.909
Asian	2	0.809(0.398–1.644)	0.558/0.001	0.881(0.546–1.423)	0.606/0.005	0.862(0.501–1.484)	0.593/0.001	0.868(0.586–1.286)	0.480/0.034
Cancer location									
Colon	5	0.786(0.537–1.152)	0.217/0.011	0.960(0.751–1.227)	0.743/<0.001	0.928(0.714–1.206)	0.578/<0.001	0.986(0.655–1.342)	0.339/0.312
Rectum	4	1.009(0.649–1.568)	0.968/0.010	0.907(0.800–1.029)	0.130/0.188	0.923(0.763–1.118)	0.413/0.062	1.052(0.747–1.481)	0.771/0.041
Source of control									
HB	4	0.918(0.543–1.554)	0.751/0.005	0.975(0.711–1.338)	0.877/0.004	0.974(0.685–1.386)	0.449/<0.001	0.933(0.705–1.384)	0.331/0.132
PB	6	0.969(0.799–1.175)	0.750/0.724	0.986(0.910–1.067)	0.722/0.149	0.984(0.912–1.062)	0.682/0.130	0.974(0.805–1.179)	0.790/0.804
HWE in controls									
Yes	8	0.981(0.815–1.181)	0.843/0.833	0.991(0.918–1.069)	0.806/0.118	0.971(0.871–1.083)	0.598/0.196	0.986(0.820–1.185)	0.881/0.909
No	2	0.809(0.398–1.644)	0.558/0.001	0.881(0.546–1.423)	0.606/0.005	0.862(0.501–1.484)	0.593/0.001	0.868(0.586–1.286)	0.480/0.034

*P* = P values for Z test. *P*_h_ = P values of Q-test for heterogeneity test. OR, odds ratio; CI, confidence intervals; HB, Hospital–based studies; PB, Population-based studies; HWE, Hardy–Weinberg equilibrium.

**Figure 2:**
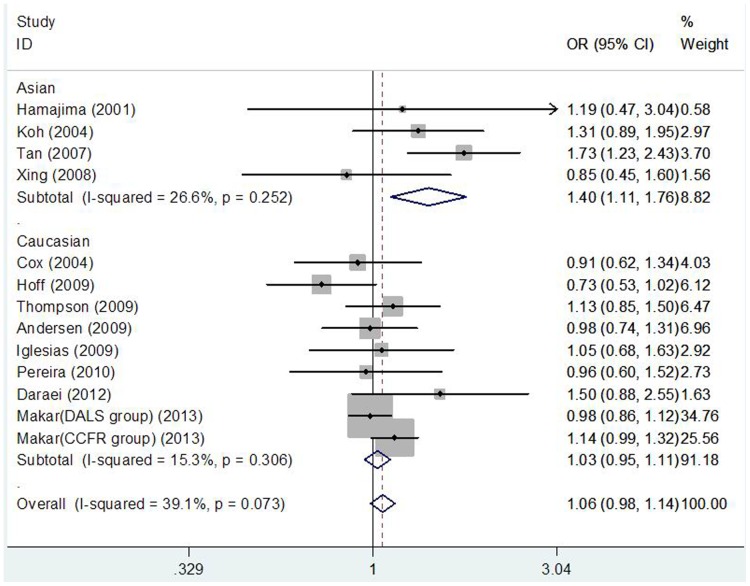
Forest plot of the COX-2 −765G>C polymorphism and CRC risk using a fixed-effect model (dominant model CC+CG vs. GG).

Table 3 lists the main results of meta-analysis of COX-2 −765G>C polymorphism and CRC risk. When all the eligible studies were pooled into the meta-analysis, statistical significant increased CRC risk was not observed in all genetic models. In subgroup analysis according to source of control, significant increased CRC risk was also not detected in hospital-based studies and population-base studies. However, in subgroup analyses by ethnicity and cancer location, after Bonferroni correction for the multiple testing (Bonferroni significance threshold P = 0.05 divided by the number of ethnicities (n = 2) or cancer types (n = 2): P = 0.025), statistical significant increased CRC risk was found in Asian populations (dominant model CC+CG vs. GG: OR = 1.399, 95%CI: 1.113–1.760, P = 0.004; Figure 2) and rectum cancer patients (CC vs. GG: OR = 2.270, 95%CI: 1.295–3.980, P = 0.004; Recessive model CC vs. CG+GG: OR = 2.269, 95%CI: 1.297–3.970, P = 0.004), but not in Caucasian populations and colon cancer patients.

**Table 3:** Meta-analysis of COX-2 −765G>C polymorphism and CRC risk.

Analysis	No. of studies	Homozygote (CC vs. GG)		Heterozygote (CG vs. GG)		Dominant model (CC+CG vs. GG)		Recessive model (CC vs. CG+GG)	
		OR (95% CI)	*P/P*_h_	OR (95% CI)	*P/P*_h_	OR (95% CI)	*P/P*_h_	OR (95% CI)	*P/P*_h_
Overall	13	1.197(0.956–1.500)	0.117/0.984	1.048(0.921–1.193)	0.477/0.031	1.072(0.958–1.200)	0.225/0.073	1.182(0.945–1.478)	0.143/0.934
Ethnicity									
Caucasian	9	1.196(0.954–1.500)	0.120/0.968	1.012(0.934–1.097)	0.767/0.168	1.027(0.950–1.111)	0.504/0.306	1.180(0.943–1.477)	0.147/0.890
Asian	4	1.420(0.788–12.931)	0.805/—	1.284(0.784–2.104)	0.320/0.131	**1.399(1.113–1.760)**	**0.004**/0.252	1.456(0.090–23.477)	0.791/—
Cancer location									
Colon	5	0.983(0.720–1.342)	0.913/0.889	1.093(0.982–1.217)	0.104/0.111	1.171(0.991–1.382)	0.063/0.085	0.966(0.709–1.317)	0.828/0.856
Rectum	4	**2.270(1.295–3.980)**	**0.004/**0.457	1.165(0.788–1.722)	0.445/0.004	1.169(0.859–1.591)	0.321/0.017	**2.269(1.297–3.970)**	**0.004/**0.588
Source of control									
HB	8	1.117(0.699–1.784)	0.643/0.938	1.058(0.811–1.381)	0.677/0.012	1.067(0.836–1.361)	0.601/0.027	1.097(0.692–1.737)	0.695/0.795
PB	5	1.223(0.946–1.582)	0.124/0.801	1.035(0.946–1.131)	0.453/0.349	1.059(0.973–1.153)	0.182/0.413	1.210(0.937–1.563)	0.144/0.766

*P* = P values for Z test. *P*_h_ = P values of Q-test for heterogeneity test. OR, odds ratio; CI, confidence intervals; HB, Hospital–based studies; PB, Population-based studies.

**Figure 3:**
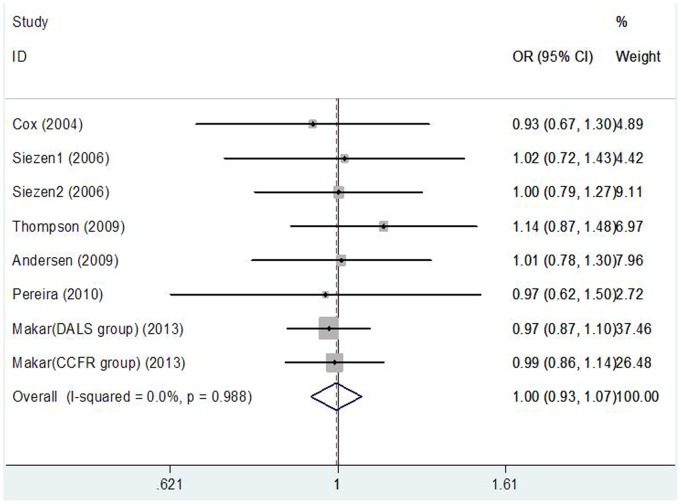
Forest plot of the COX-2 8473T>C polymorphism and CRC risk using a fixed-effect model (dominant model CC+CT vs. TT).

Table 4 lists the main results of meta-analysis of COX-2 8473T>C polymorphism and CRC risk. There was no evidence of significant association between COX-2 8473T>C polymorphism and CRC risk when all eligible studies were pooled into the meta-analysis (CC vs. TT: OR = 0.948, 95%CI: 0.843–1.066, P = 0.369; TC vs. TT: OR = 1.008, 95%CI: 0.934–1.088, P = 0.841; CC+TC vs. TT: OR = 0.995, 95%CI: 0.926–1.070, P = 0.899, Figure 3; CC vs. TC+TT: OR = 0.941, 95%CI: 0.842–1.051, P = 0.284). In subgroup analyses by ethnicity, cancer location, and source of controls, statistical significant association was also not observed in all subgroups.

**Table 4:** Meta-analysis of COX-2 8473T>C polymorphism and CRC risk.

Analysis	No. of studies	Homozygote (CC vs. TT)		Heterozygote (TC vs. TT)		Dominant model (CC+TC vs. TT)		Recessive model (CC vs. TC+TT)	
		OR (95% CI)	*P/P*_h_	OR (95% CI)	*P/P*_h_	OR (95% CI)	*P/P*_h_	OR (95% CI)	*P/P*_h_
Overall	8	0.948(0.843–1.066)	0.369/0.824	1.008(0.934–1.088)	0.841/0.969	0.995(0.926–1.070)	0.899/0.988	0.941(0.842–1.051)	0.284/0.713
Ethnicity									
Caucasian	8	0.948(0.843–1.066)	0.369/0.824	1.008(0.934–1.088)	0.841/0.969	0.995(0.926–1.070)	0.899/0.988	0.941(0.842–1.051)	0.284/0.713
Cancer location									
Colon	3	0.923(0.787–1.083)	0.326/0.336	1.014(0.911–1.129)	0.795/0.574	0.994(0.898–1.099)	0.902/0.471	0.916(0.788–1.064)	0.250/0.382
Rectum	2	0.946(0.726–1.233)	0.682/0.554	1.020(0.861–1.208)	0.823/0.748	1.005(0.855–1.180)	0.955/0.642	0.937(0.729–1.203)	0.609/0.606
Source of control									
HB	2	0.995(0.620–1.597)	0.984/0.788	0.932(0.706–1.231)	0.622/0.801	0.943(0.724–1.230)	0.667/0.896	1.030(0.655–1.620)	0.899/0.724
PB	6	0.945(0.837–1.066)	0.356/0.626	1.014(0.937–1.098)	0.727/0.921	1.000(0.927–1.077)	0.950/0.992	0.936(0.835–1.049)	0.255/0.510

*P* = P values for Z test. *P*_h_ = P values of Q-test for heterogeneity test. OR, odds ratio; CI, confidence intervals; HB, Hospital–based studies; PB, Population-based studies.

### Heterogeneity analysis

For the COX-2 −1195A>G polymorphism, statistical significant heterogeneity among studies was observed when all eligible studies were pooled into the meta-analysis (GG vs. AA: P_h_ = 0.022; AG vs. AA: P_h_ = 0.004; GG+AG vs. AA: P_h_<0.0001). To explore the sources of heterogeneity, we first performed subgroup analyses. Subgroup analyses by ethnicity, cancer location, and source of controls showed that the heterogeneity was still significant in Asian populations, hospital-based studies, colon cancer patients and rectum cancer patients. Subsequently, we performed meta-regression analysis to further identify the source of heterogeneity. Meta-regression analysis indicated that the HWE in controls was the major source which contributed to heterogeneity. When we excluded two HWE-violating studies [16], [32], the heterogeneity disappeared (GG vs. AA: P_h_ = 0.833; AG vs. AA: P_h_ = 0.118; GG+AG vs. AA: P_h_ = 0.196). However, the significance of the summary ORs for COX-2 −1195A>G polymorphism in different comparison models were not influenced by omitting the two studies (Table 2).

For the COX-2 −765G>C and 8473T>C polymorphisms, statistical significant heterogeneity was not detected in the overall populations and subgroup analyses.

### Sensitivity analysis

Sensitivity analysis was performed by sequential omission of individual studies. For all these three polymorphisms (−1195A>G, −765G>C, and 8473T>C), the significance of pooled ORs under all contrast models in both total population and subgroup analyses was not influenced excessively by omitting any single study (data were not shown).

For −1195A>G polymorphism, sensitivity analysis was further performed by omitting those two studies [16], [32] in which genotype distribution of −1195A>G polymorphism in the controls were significantly deviated from HWE. The significance of pooled ORs in both total population and subgroup analyses was not influenced by omitting these two studies.

### Publication bias

**Figure 4:**
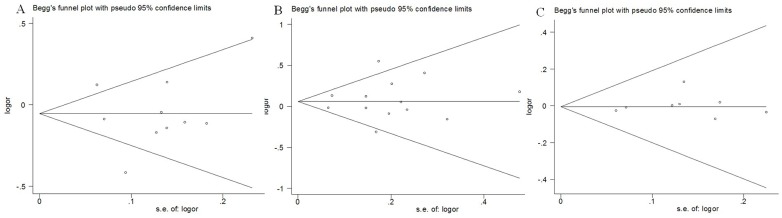
Funnel plot analysis to detect publication bias. Each point represents a separate study for the indicated association. **A** Funnel plot for dominant model GG+AG vs. AA of COX-2 −1195 G>A polymorphism in overall analysis (P = 0.890); **B** Funnel plot for dominant model CC+CG vs. GG of COX-2 −765G>C polymorphism in overall analysis (P = 0.703); C Funnel plot for dominant model CC+CT vs. TT of COX-2 8473T>C polymorphism in overall analysis (P = 0.518).

Begg's funnel plot and Egger's test were performed to assess the publication bias of literatures in all comparison models. The shape of the funnel plot did not reveal any evidence of obvious asymmetry. Then, the Egger's test was used to provide statistical evidence of funnel plot symmetry. The results still did not suggest any evidence of publication bias in −1195A>G (*P* = 0.330 for GG vs. AA; *P* = 0.853 for AG vs. AA; *P* = 0.312 for recessive model GG vs. AG+AA; and *P* = 0.890 for dominant model GG+AG vs. AA), −765G>C (*P* = 0.332 for CC vs. GG; *P* = 0.815 for CG vs. GG; *P* = 0.389 for recessive model CC vs. CG+GG; and *P* = 0.703 for dominant model CC+CG vs. GG), and 8473T>C (*P* = 0.376 for CC vs. TT; *P* = 0.921 for TC vs. TT; *P* = 0.423 for recessive model CC vs. TC+TT; and *P* = 0.518 for dominant model CC+TC vs. TT) polymorphisms; Figure 4.

## Discussion

The present meta-analysis, including 18,702 cases and 27,311 controls from 16 case–control studies, was conducted systematically to evaluate the association between the genetic variants in the COX-2 gene and CRC risk. To our knowledge, this is the most comprehensive meta-analysis to date to evaluate the association between COX-2 polymorphisms and CRC risk. Our results showed that the COX-2 −765G>C polymorphism was associated with an increased CRC risk among Asians (dominant model CC+CG vs. GG: OR = 1.399, 95%CI: 1.113–1.760, P = 0.004), which was in accordance with the previously published meta-analysis by Cao et al. [13]. However, with respect to −1195A>G and 8473T>C polymorphisms, no significant association with CRC risk was demonstrated in the overall and subgroup analyses.

This finding may be biologically plausible. Cyclooxygenases are central enzymes in the prostaglandin pathway that convert free arachidonic acid into the intermediate prostaglandin H2 which is the precursor of prostaglandins, prostacyclin, and thromboxanes. Currently, three COX isoenzymes were reported: COX-1, COX-2, and COX-3 [39]. COX-2 is normally absent in most cells and tissues. It was induced in response to inflammatory cytokines, hypoxia, mitogens, hormones, angiogenic growth factors, and tumor promoters [40]. COX-2-derived prostaglandins, prostacyclin, and thromboxanes participate in many biologic processes such as apoptosis inhibition, inflammation, immune response suppression, tumor cell invasion, metastasis, and angiogenesis, which are all crucial in the development and progression of cancer [39], [41], [42]. It was shown that polymorphisms in the promoter of COX-2 may exert profound effects on gene transcriptional activity by altering the binding capacity of certain nuclear proteins, there-by affecting expression of COX-2 enzyme [43]. COX-2 −765G>C is a functional polymorphism located at 765 bp upstream (−765 bp) from the transcription starting site. It changes a putative stimulatory protein (Sp1) binding site in the promoter of COX-2 between −766 and −761 bp [44], but it creates an E2 promoter factor (E2F) binding site, leading to high transcription activity and increased COX-2 expressions which might be involved in the development of cancers [45]. More importantly, the homozygous variant genotype COX-2 −765CC has been shown associated with increased risk for many different types of cancers, including breast cancer [46], ovarian cancer [47], hepatocellular carcinoma [48] and lung cancer [49].

In subgroup analysis by ethnicity, the COX-2 −765G>C polymorphism presented a risk factor for CRC in Asian populations, but not in Caucasians. The inconsistent data among the different ethnicities may indicate different effects of the COX-2 −765G>C polymorphism on CRC risk in different ethnic genetic backgrounds. Nevertheless, owing to the limited number of relevant studies among Asian population included in this meta-analysis, the observed positive association between COX-2 −765G>C polymorphism and CRC risk in Asians is likely to be caused by chance because study with small sample sizes may have insufficient statistical power to detect a slight effect or may have generated a fluctuated risk estimate. Currently there are only 4 studies on COX-2 −765G>C polymorphism and CRC risk among Asian population [28], [30], [32], [33]. Therefore, the positive results of the Asain population should be interpreted with caution.

Studies have suggested that cancers of the rectum and colon might be distinct tumors because they have a differing prevalence with a difference in clinical presentation, prognosis and possibly in genetic and environmental epidemiology. Thus, the COX-2 −765G>C polymorphism might influence carcinogenesis of colorectal tissues in a site-specific manner. Therefore, we carried out subgroup analysis according to cancer location. Our results suggested a significant increased CRC risk in rectum cancer patients (CC vs. GG: OR = 2.270, 95%CI: 1.295–3.980, P = 0.004; Recessive model CC vs. CG+GG: OR = 2.269, 95%CI: 1.297–3.970, P = 0.004) but not in colon cancer subjects, which was consistent with the results of the large sample study by Markar et al. [14]. Our findings add further data to evidence that colon and rectal cancers have different etiologies.

In our meta-analysis, several limitations should be acknowledged. First, in subgroup analyses by ethnicity and cancer location, the sample size of population was relatively small for subgroup analyses, which may lead to relatively weak power to detect the real relationship. Second, our results were based on unadjusted estimates. We did not perform the analysis adjusted for other covariates such as smoking, drinking, obesity, red meat consumption, and so on, because of the unavailable original data of the eligible studies.

In conclusion, our meta-analysis provided a more precise estimation based on larger sample size compared with the individual studies and previous meta-analysis. Our study suggested that COX-2 −765G>C polymorphism might contribute to colorectal cancer risk, especially in Asian populations and the rectum cancer patients. In order to further verify our findings, large well designed epidemiological studies are warranted.

## Supporting Information

Figure S1 **Flow diagram of included studies for this meta-analysis.**
(TIF)

Checklist S1 (DOC)
