# Supplementary figures and images for: Meta-Analysis of the Association between COX-2 Polymorphisms and Risk of Colorectal Cancer Based on Case–Control Studies
Source: PLoS One. 2014 Apr 14;9(4):e94790. doi: 10.1371/journal.pone.0094790 (PMC3986224; doi:10.1371/journal.pone.0094790)

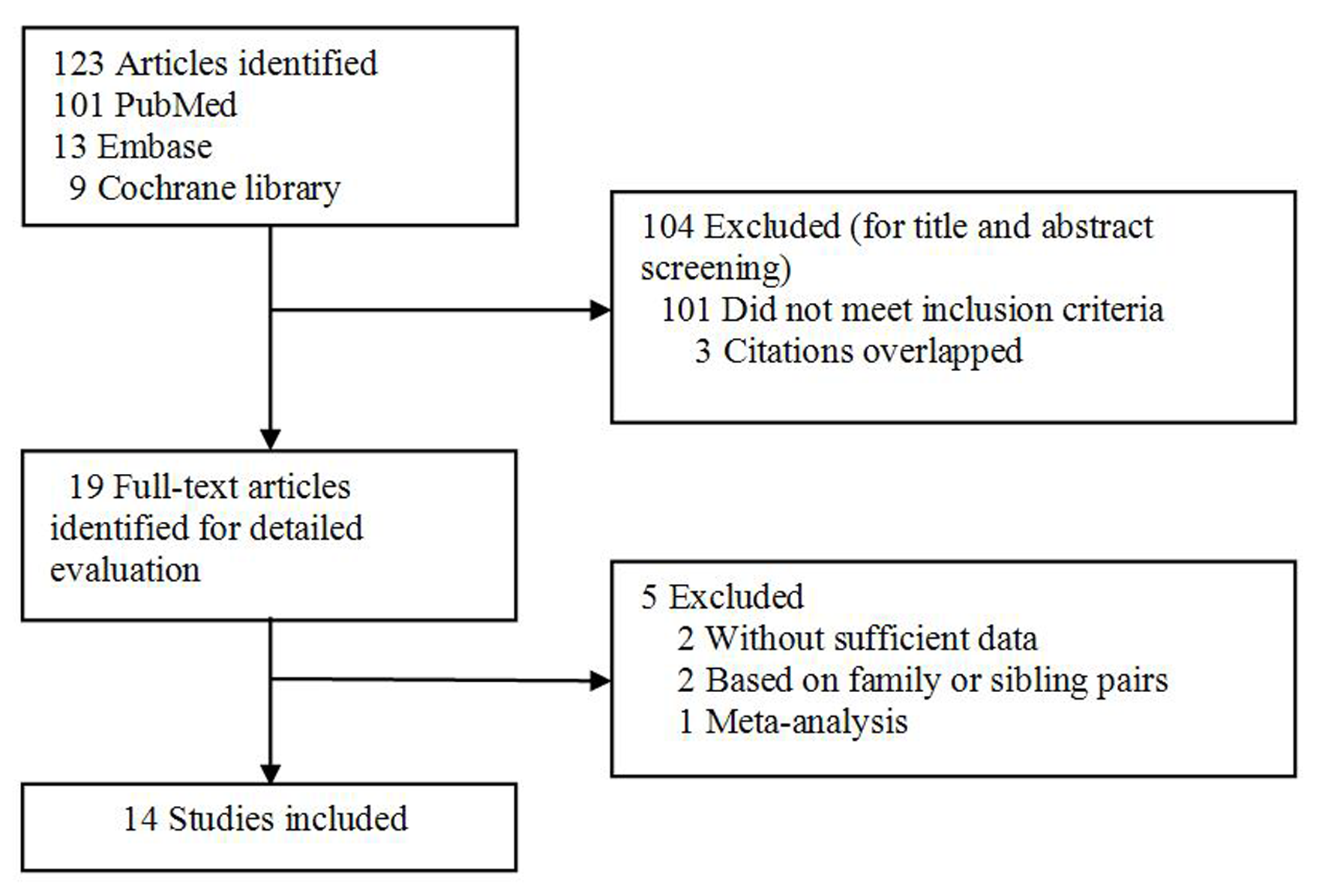

Supplement: Figure S1 — Flow diagram of included studies for this meta-analysis. (TIF) [file pone.0094790.s001.tif]
